# Virulence and antimicrobial resistance characteristics of *Vibrio parahaemolyticus* isolated from environment, food and clinical samples in the south of Vietnam, 2010

**Published:** 2011-01-10

**Authors:** Diep The Tai, Au Vinh Thuy, Nguyen Thi Ngoc Nhi, Nguyen Thi Kim Ngoc, Nguyen Thi Phuong Lan

**Affiliations:** 1 Pasteur Institute of Ho Chi Minh City, Vietnam

## Background

*Vibrio parahaemolyticus* is a major cause of food poisoning in many countries. The rapid cases of this agent in recent years made it become an important etiology. However, the relationship between strains isolated from environment, food and clinical samples is still unclear. The presence of antimicrobial resistance and virulence factors such as tdh (thermostable direct hemolysin), trh (the TDH – related hemolysin) are proven to be candidate markers for studying on *Vibrio parahaemolyticus*. In Vietnam, the existence of *Vibrio parahaemolyticus* is poorly understood. Our objective was to analyse the presence of *Vibrio parahaemolyticus* from different sources of samples as well as its virulent factors (tdh and trh genes) and antibiotic resistance characteristics.

## Methods

817 samples (food: 85, water: 299, stool of acute diarrhea: 433) were collected from May to July, 2010 at Ho Chi Minh City, Can Tho City, Ca Mau, Ben Tre, An Giang and Bac Lieu provinces from Southern of Vietnam. All samples were cultured and bacterial identified by API 20E. Besides, PCR for ToxR gene was performed for confirmation of *Vibrio parahaemolyticus.* The presence of either tdh or trh gene were done as described previously. Diffusion agar method and MIC were used for screening the antibiotic susceptibility of all identified strains.

## Results

15.91 % (130/817) *Vibrio parahaemolyticus* was isolated including 8.3% (36/434) from acute diarrheal patients, 40% (34/85) from foods and 20.1% (60/299) from environment samples. All strains had ToxR gene, but not for tdh and trh genes. In patient, just only 22.2% (8/36) strains had tdh gene and 19.4 % (7/36) strains got trh gene. All isolated strains from food were negative with both of tdh and trh gene. To trains isolated from environment, the presence of trh gene is 33.3% (20/60) and all of them got negative with tdh gene. To susceptibility test, most of strains was sensitive with tetracycline (90.77%, MIC ≥ 0.5 μg/ml), chloramphenicol (97, 69%, MIC ≥ 4 μg/ml ), ciprofloxacin (100%, MIC ≥ 0.125 μg/ml), bactrim, doxycycline (93.08%) and resistance with ampicillin (34.62%, MIC ≥ 16 μg/ml), tetracycline and bactrim (6.92%), chloramphenicol, doxycycline (2.31% and 4.62% ) respectively; reduced susceptibility was detected in *Vibrio parahaemolyticus* for ampicillin (45.38%), tetracycline, doxycycline (2.31%).

## Conclusion

The circulation of *Vibrio parahaemolyticus* was quite high in the south of Vietnam. Virulence genes (tdh, trh) are not the only factors to cause diarrhea, we need to find another virulence factors. Most of isolated strains were sensitive with antibiotic except ampicillin.

